# Exercise transcutaneous oxygen pressure measurement: From a small research field to a worldwide community clinical practice

**DOI:** 10.1113/EP092877

**Published:** 2025-05-27

**Authors:** Marjolaine Talbot, Adelaïde Guézais, Guillaume Mahé

**Affiliations:** ^1^ CHU Rennes, Vascular Medicine Unit Rennes France; ^2^ CHU Rennes, Inserm, CIC 1414 (Clinical Investigation Center) Rennes France; ^3^ Univ Rennes Rennes France

**Keywords:** Oximetry, peripheral artrey disease

1

Lower extremity peripheral artery disease (PAD) has a global prevalence of more than 200 million patients, of whom approximately half exhibit symptoms of limb discomfort during walking activity. The diagnosis of PAD is based on several means such as ankle‐brachial index, Doppler waveforms analysis or toe‐brachial index, which are measured at rest (Mahé et al., 2021). In the event of inconclusive results from measurements taken at rest, other means measured during exercise or after exercise may be utilized. Transcutaneous oxygen pressure (TcPO_2_) measurement, which was previously used to diagnose critical limb ischaemia, a condition in which patients have insufficient blood supply at rest, is also one of the means that can be used during exercise. Abraham et al. (2003) were the first to propose a novel parameter to detect arterial stenosis of the lower limbs using Exercise‐TcPO_2_ (Abraham et al., 2003). This parameter is called either the delta from rest oxygen pressure or decrease of rest oxygen pressure (DROP) and has a good sensitivity ranging from 79% to 86% and specificity ranging from 76% to 86% as reported by several teams to detect arterial stenosis >70–75% (Abraham et al., 2003; Mahé et al., 2021; Stivalet et al., 2019). In the present issue of *Experimental Physiology*, the team led by Prof Abraham (France) summarized the development of Exercise‐TcPO_2_ and the enhancement of the technique since 2003 by analysing their patient database containing more than 9000 patients (Lecoq et al., 2025). Even if the technique appears promising and quite easy to perform there are still several challenges.

The first one is the absence of commercially available software to calculate the DROPs at each site in real‐time. As mentioned in their paper, Lecoq and colleagues use in‐house software that analyses the data recorded by Radiometer and Perimed systems, which are approved by the Food and Drug Administration (FDA) and marked by the European Commission (CE). On our side, we are using Oxymonitor software that is also in‐house software that we validated and which can be downloaded for free (https://imagemed.univ‐rennes1.fr/en/oxymonitor/download) (Mahé et al., 2021; Poulin et al., 2018). Unfortunately, to date, none of this software is FDA approved or CE marked. Thus, we call on manufacturers to develop approved software. Readily available and approved software will help to answer the second challenge.

The second challenge is the inadequate level of attention paid to the technique in the international guidelines (Mahé et al., 2021), despite improvements having been mentioned by Lecoq et al. (2025). The French guidelines only suggest the use of the technique as follows: ‘In the event of difficulty in diagnosing or excluding LEAD, we suggest proposing the measurement of Exercise‐TcPO_2_ in patients with complicated pathological conditions (e.g. diabetes, lumbar spinal canal stenosis) (grade 2^+^)’ (Mahé et al., 2021).There are several explanations for this lack of attention. (i) It was thought to be a technique used by only a few physicians. However, it is clear that the technique has been spreading for 4–5 years and that the technique is no longer confined to a small community in the research field (Figure 1). (ii) It was considered no more informative than post‐exercise ankle brachial index which was cheaper and less time‐consuming. Yet, a number of observational studies have demonstrated the value of post‐exercise and Exercise‐TcPO_2_ in clinical practice with a possible interest of the combination of the tests. And (iii) no randomized controlled trial has been performed to demonstrate the interest of the Exercise‐TcPO_2_ for PAD patient management resulting in the absence or low reimbursement of this technique by the authorities. Indeed, in the section ‘Directions for the future’, Lecoq et al. raised the questions of which population should undergo this procedure and whether there is an interest in using Exercise‐TcPO_2_ to adapt rehabilitation programmes or estimate the results of medical or surgical treatments.

**FIGURE 1:**
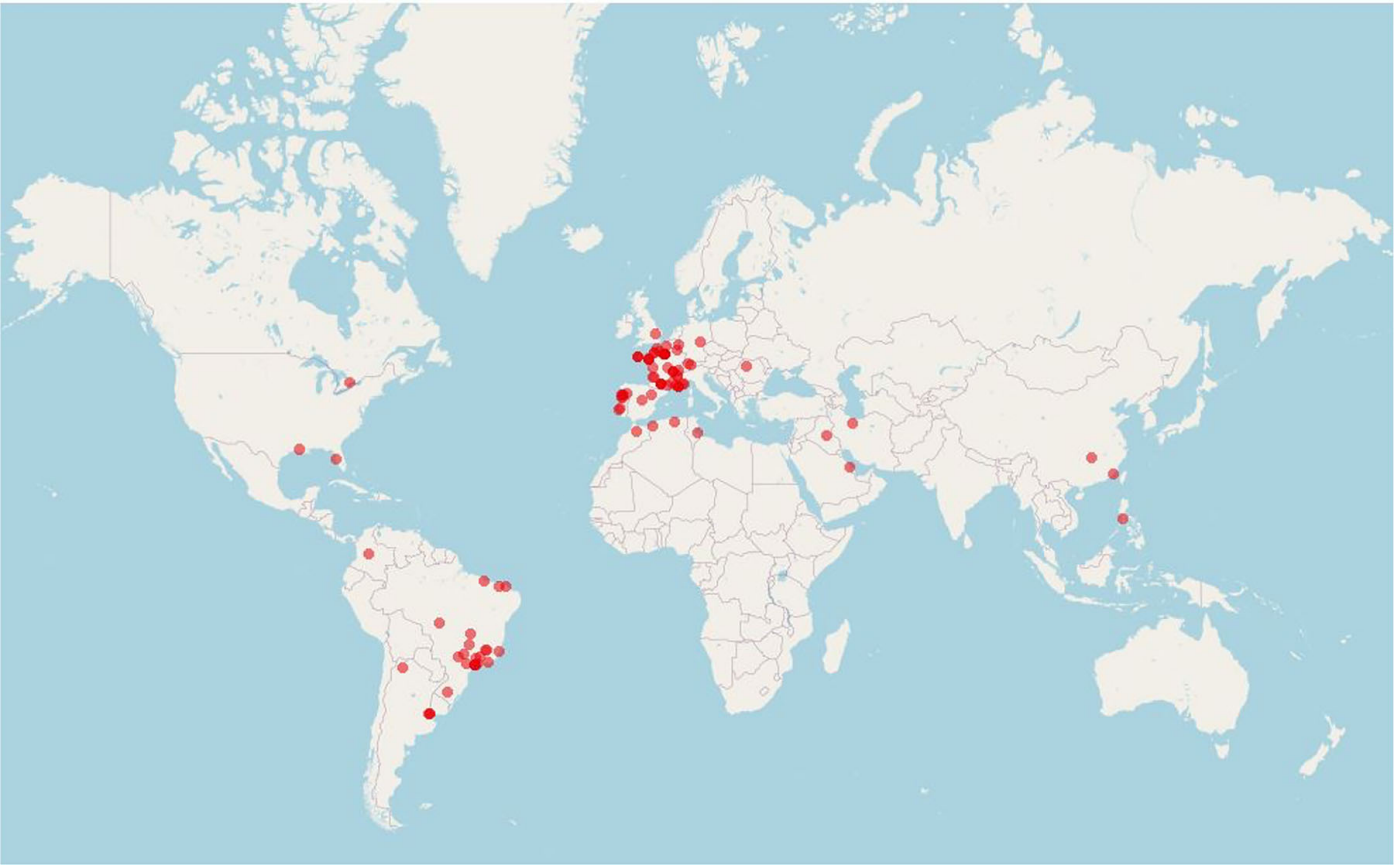
Sites which have downloaded the Oxymonitor software.

Thus, we invite you to read the paper in the present issue of *Experimental Physiology* and contribute to the development of the use of Exercise‐TcPO_2_ in research on the physiology and vascular medicine fields and finally in clinical practice.

## AUTHOR CONTRIBUTIONS

All authors have approved the final version of the manuscript and agree to be accountable for all aspects of the work in ensuring that questions related to the accuracy or integrity of any part of the work are appropriately investigated and resolved. All persons designated as authors qualify for authorship, and all those who qualify for authorship are listed.

## CONFLICT OF INTEREST

None declared.

## FUNDING INFORMATION

No funding was received for this work.
